# Visible-Light-Driven Photocatalysis of Carbon Dioxide and Organic Pollutants by CaBiO_2_Cl/g-C_3_N_4_

**DOI:** 10.3390/molecules30183760

**Published:** 2025-09-16

**Authors:** Yu-Yun Lin, Bo-Heng Huang, Wen-Yu You, Fu-Yu Liu, Jia-Hao Lin, Chiing-Chang Chen

**Affiliations:** Department of Science Education and Application, National Taichung University of Education, Taichung 403514, Taiwan; cuteyylin@gmail.com (Y.-Y.L.); asc109124@gm.ntcu.edu.tw (B.-H.H.); asc106131@gm.ntcu.edu.tw (W.-Y.Y.); nancy50851@gmail.com (F.-Y.L.); bsc104103@gm.ntcu.edu.tw (J.-H.L.)

**Keywords:** perovskite-type, photocatalyst, Rhodamine 6G, CO_2_ reduction

## Abstract

Perovskite-type CaBiO_2_Cl with a unique layered Sillen X1 structure exhibits great potential as an efficient visible-light photocatalyst. In this study, CaBiO_2_Cl was synthesized through calcination at 800 °C and subsequently composited with varying amounts of g-C_3_N_4_ to optimize photocatalytic performance. The prepared catalysts were characterized by multiple techniques to confirm their structural and compositional features. Under visible-light irradiation, the photocatalytic activities toward Rh6G degradation were systematically evaluated using UV–vis PDA and EPR analyses. To further elucidate the degradation mechanism, radical scavenger experiments were conducted to identify the reactive species generated during the photodegradation process. Kinetic analysis revealed that the reaction rate constant (*k*) of pure CaBiO_2_Cl was 0.0525 h^−1^, while that of pure g-C_3_N_4_ was 0.0423 h^−1^. Notably, the CaBiO_2_Cl/10 wt% g-C_3_N_4_ composite exhibited an enhanced *k* value of 0.0568 h^−1^, which is 1.1 and 1.3 times higher than those of CaBiO_2_Cl and g-C_3_N_4_, respectively. Furthermore, under ambient conditions (25 °C, 1 atm), the CO_2_-to-CH_4_ photocatalytic conversion efficiency of the CaBiO_2_Cl/10 wt% g-C_3_N_4_ composite reached 0.5652 μmol g^−1^ h^−1^. These findings demonstrate that CaBiO_2_Cl-based composite photocatalysts not only achieve superior visible-light photocatalytic activity but also exhibit excellent stability, highlighting their potential for environmental remediation and alignment with the principles of green chemistry.

## 1. Introduction

Over the past three decades, semiconductor photocatalysts have emerged as a research focus in the fields of renewable energy conversion and environmental remediation. From the perspective of solar energy utilization, a key challenge lies in developing materials with strong absorption and catalytic activity under visible light. Common strategies include anion substitution to tailor the electronic band structure—raising the valence band maximum and narrowing the bandgap [1] and doping visible-light-inactive metal oxides with foreign elements to extend their absorption range [2]. However, dopant-induced defect states often serve as charge recombination centers, thereby suppressing photocatalytic performance [3]. In contrast, non-oxide materials, such as oxynitrides and oxysulfides, which inherently contain nitrogen or sulfur atoms with high-energy p-orbitals, exhibit both elevated valence band positions and enhanced visible-light absorption while maintaining favorable charge transport properties [4].

Among visible-light-responsive photocatalysts, BiOX (X = Cl, Br, I) has attracted widespread attention due to its unique layered crystal structures and excellent optoelectronic properties [5]. BiOX compounds possess a typical two-dimensional Sillén-type layered structure that leads to strong anisotropy in their electrical and optical behaviors—features that render them promising for photocatalysis and optoelectronic applications [6]. The internal electric field generated within their layered structures facilitates efficient separation of photogenerated electron–hole pairs, thus improving photocatalytic activity under visible-light irradiation [7].

Structurally, BiOX consists of [Bi_2_O_2_]^2+^ slabs separated by halide ions (X^−^ = Cl^−^, Br^−^, I^−^), forming a Sillén X2-type structure [8]. Similar arrangements are found in (Bi,M)_2_O_2_X compounds, where partial substitution of Bi^3+^ by monovalent or divalent metals (M = Na, Sb, Cd, Pb, Sr, Ca, Ba or Li) yields alternating layers of [(Bi,M)_2_O_2_]^2+^ and halide anions, known as the Sillén X1-type structure [9,10]. Such structural modifications provide a feasible route for band structure tuning and visible-light activity enhancement. Due to their layered lattices and high chemical stability, bismuth-based oxyhalides are increasingly considered promising photocatalyst candidates [11]. Among them, CaBiO_2_Cl stands out as a representative Sillén X1-type material [12], comprising [CaBiO_2_]^+^ layers with a fluorite-like motif and interleaved single Cl^−^ anion layers [13]. Its highly ordered structure and strong photoluminescence have attracted growing research interest. Although recent studies have reported its photocatalytic activity in methylene blue (MB) dye degradation, a comprehensive understanding of its photophysical properties and reaction mechanisms remains limited [12].

With continued technological advancement and rising demand for quality of life, synthetic dyes are produced and consumed on a massive scale, contributing significantly to environmental pollution. More than 1.2 million tons of dyes are manufactured annually, with large quantities released into air, water, and soil systems. Triphenylmethane dyes, prized for their vibrant colors and stability, are widely used in the textile industry but are also of environmental concern due to their toxicity and poor biodegradability, leading many countries to restrict or ban their use. Nonetheless, these dyes remain in use due to continued market demand. It is estimated that 10–20% of dye-containing wastewater is discharged untreated, making dye pollution a pressing environmental issue. Various treatment technologies have been developed, including adsorption [14], biodegradation [15] and photocatalysis [16]. Among these, semiconductor photocatalysis is particularly appealing due to its high efficiency, solar-driven mechanism, and environmental friendliness. Photocatalytic reactions operate under mild conditions, converting solar energy into chemical energy and efficiently decomposing organic pollutants.

In this work, we systematically investigate the photodegradation performance of CaBiO_2_Cl and CaBiO_2_Cl/g-C_3_N_4_ using Rhodamine 6G (Rh6G). Rh6G is currently widely encapsulated in nanoparticles for applications like cellular labeling and intraocular cell tracking, and it is also frequently employed in intraocular surgical procedures. Colorimetric assays have demonstrated that Rh6G exhibits significant ocular cytotoxicity toward ARPE-19 cells, even at concentrations as low as 0.2% or higher. However, no in vivo toxicity assessments have been reported to date [17]. It is already established that rhodamine and its derivatives pose potential health hazards, including toxicity upon ingestion and irritation to the skin, eyes, and respiratory tract [18].

Beyond dye degradation, this study also explores the potential of CaBiO_2_Cl and CaBiO_2_Cl/g-C_3_N_4_ for photocatalytic CO_2_ reduction to hydrocarbon fuels. To date, no research has reported the use of CaBiO_2_Cl/g-C_3_N_4_ in photocatalytic CO_2_ conversion, highlighting a gap that this work aims to address. Against the backdrop of global climate change and the energy crisis, excessive CO_2_ emissions have become a leading cause of global warming [19]. As such, developing carbon-neutral technologies has become a scientific imperative. Photocatalytic CO_2_ reduction, which mimics natural photosynthesis, is a promising strategy that utilizes solar energy to convert CO_2_ into value-added products, such as CO, CH_4_, and CH_3_OH. This approach not only mitigates greenhouse gas levels but also enables carbon recycling and renewable fuel production [20].

To enhance the performance of the photocatalyst, this study adopts the strategy of compounding graphitic carbon nitride (g-C_3_N_4_) with our materials. In recent years, graphitic carbon nitride has emerged as a promising metal-free photocatalyst due to its moderate bandgap (~2.7 eV), good thermal stability, and visible light responsiveness [21]. However, the pristine form of g-C_3_N_4_ suffers from rapid recombination of photogenerated electron–hole pairs, low surface area, and limited light absorption, which significantly restrict its photocatalytic performance in CO_2_ reduction and organic pollutant degradation [22]. To address these limitations, constructing heterojunctions by coupling g-C_3_N_4_ with other semiconductors [23,24,25,26], carbon-based materials (e.g., graphene, reduced graphene oxide) [27], or cocatalysts has been widely explored [28]. These hybrid systems can effectively enhance charge separation, extend light absorption into the visible region, and increase the number of surface active sites [29]. In CO_2_ photoreduction, such modifications improve the selectivity and yield of target products like CO or CH_4_ [30]. For dye degradation, the synergistic effect at the heterojunction interface facilitates the generation of reactive oxygen species (ROS) and accelerates the photocatalytic oxidation process [31]. Overall, the rational design of g-C_3_N_4_-based composite photocatalysts offers an effective strategy to enhance both the activity and stability of solar-driven environmental and energy-related applications.

Compared to conventional thermocatalytic routes, photocatalytic systems offer the advantages of ambient reaction conditions, low energy input, and use of solar energy as a renewable driving force [32]. These features make them particularly attractive for integrating solar-to-fuel technologies. Although current CO_2_ reduction photocatalysis faces challenges, such as low efficiency [33], limited selectivity [34], and product separation [35], continued advances in catalyst design, mechanistic understanding, and system integration may unlock its full potential for sustainable applications.

## 2. Results and Discussion

### 2.1. As-Prepared Sample Characterization

#### 2.1.1. Powder XRD

**Figure 1:**
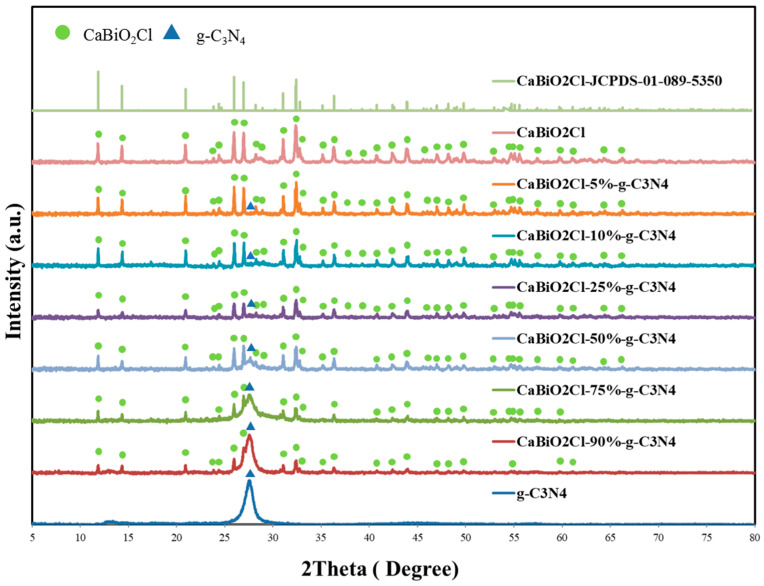
XRD patterns of as-prepared CaBiO_2_Cl/g-C_3_N_4_ samples.

Prior to the preparation of the CaBiO_2_Cl/g-C_3_N_4_ composite photocatalyst, XRD analyses were performed on CaBiO_2_Cl and g-C_3_N_4_ before and after use. The diffraction peaks of CaBiO_2_Cl matched well with the JCPDS card No. 01-089-5350, while a characteristic peak of g-C_3_N_4_ appeared at 2θ = 27.4° [36]. As shown in Figure 1, with the increasing content of g-C_3_N_4_ in CaBiO_2_Cl, the characteristic peak of g-C_3_N_4_ gradually intensified, whereas the diffraction peak intensity of CaBiO_2_Cl correspondingly decreased. Notably, no new crystalline phases were detected, indicating that the introduction of g-C_3_N_4_ primarily occurred in the form of surface loading or coupling without altering the crystal structure of CaBiO_2_Cl. In addition, the absence of extra peaks in the XRD patterns further excluded the formation of secondary phases or impurities during the synthesis. These results demonstrate that the CaBiO_2_Cl/g-C_3_N_4_ composite possesses excellent structural stability, providing a reliable structural basis for its subsequent photocatalytic applications.

#### 2.1.2. FT-IR

**Figure 2:**
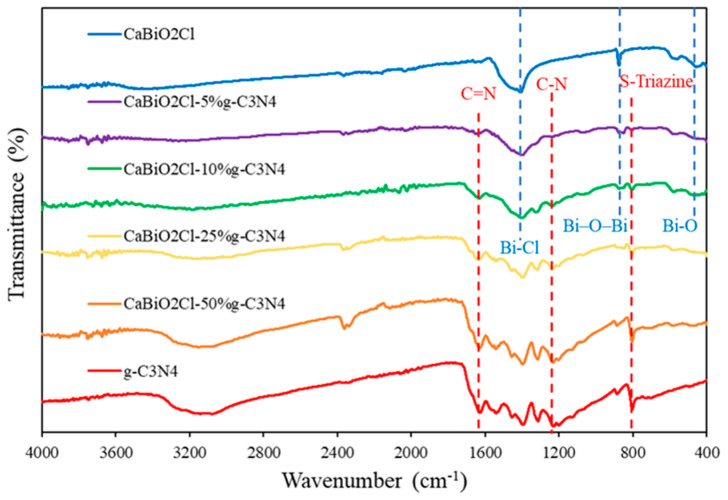
FT−IR spectra of the as-prepared CaBiO_2_Cl/g-C_3_N_4_.

In Figure 2, the characteristic vibrational absorption peaks of CaBiO_2_Cl are observed at 527 cm^−1^ (Bi–O), 840 cm^−1^ (Bi–O–Bi) [37], and 1458 cm^−1^ (Bi–Cl) [38]. In addition, the absorption features of g-C_3_N_4_ are clearly identified; the band at 809 cm^−1^ corresponds to the breathing mode of the 1,3,5-triazine ring, the peak near 1642 cm^−1^ is assigned to C=N stretching, while the bands at 1241, 1319, and 1409 cm^−1^ are attributed to C–N stretching vibrations [39]. For the CaBiO_2_Cl/g-C_3_N_4_ composites, the characteristic peaks of g-C_3_N_4_ gradually intensify with increasing g-C_3_N_4_ content, whereas those of CaBiO_2_Cl exhibit slight shifts and intensity variations, indicating strong interfacial interactions between the two components. FTIR spectroscopy thus confirms the successful incorporation of g-C_3_N_4_ into CaBiO_2_Cl at different proportions.

#### 2.1.3. FE-SEM-EDS

**Figure 3:**
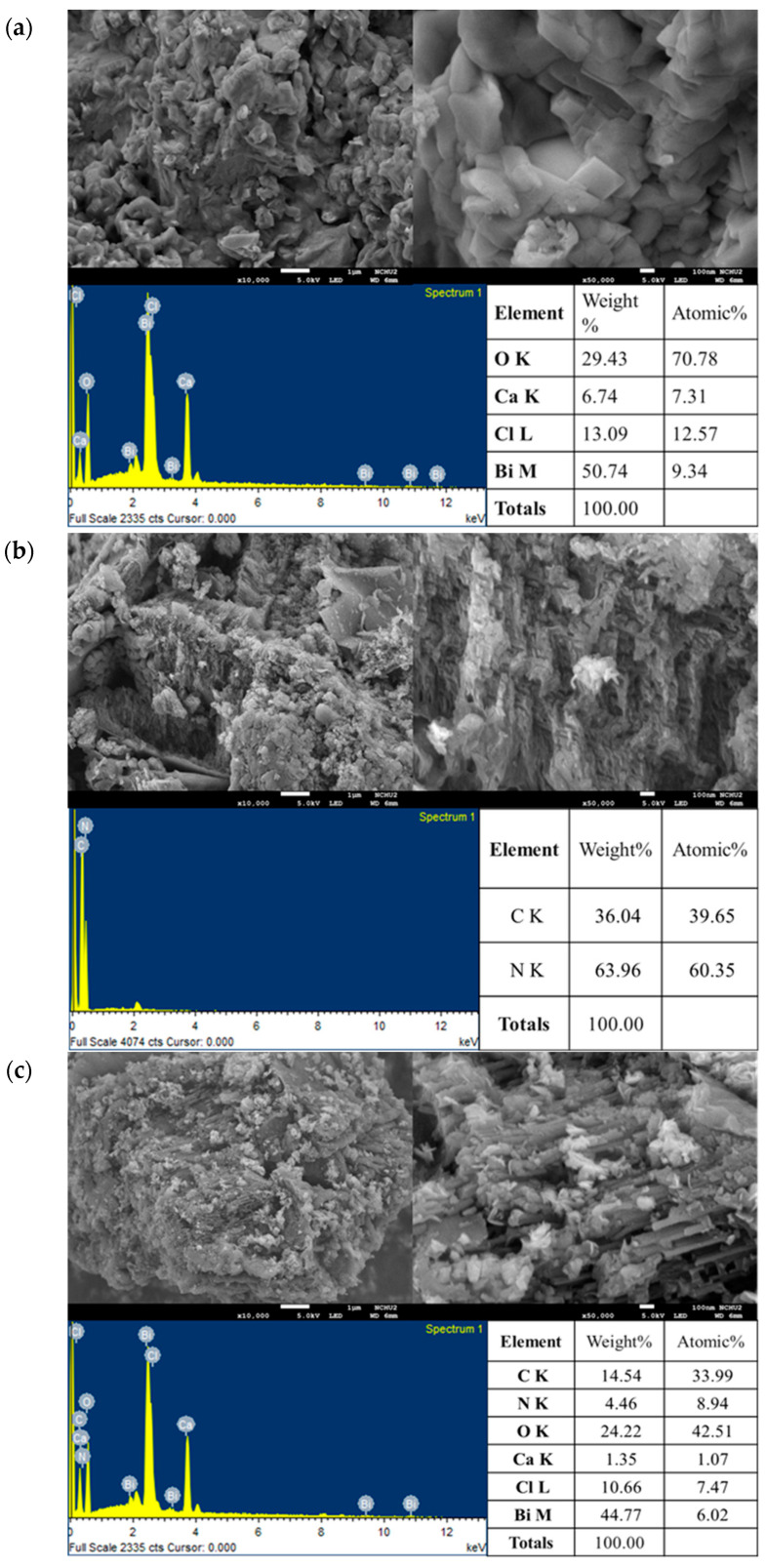
SEM images 10,000×, 50,000×, and EDS of the (**a**) CaBiO_2_Cl, (**b**) g-C_3_N_4_, and (**c**) CaBiO_2_Cl/10 wt% g-C_3_N_4_ samples.

Figure 3a, Figure 3b and Figure 3c show the FE-SEM images of the CaBiO_2_Cl photocatalyst, pure g-C_3_N_4_, and the CaBiO_2_Cl/10 wt% g-C_3_N_4_, respectively, at magnifications of 10,000× and 50,000×. The morphology of the CaBiO_2_Cl sample appears as small stacked lamellar structures, while pure g-C_3_N_4_ exhibits a sheet-like structure. The composite sample retains features of both materials. Furthermore, semi-quantitative elemental analysis using EDS confirms the presence of C, N, O, Ca, Cl, and Bi in the samples, with their atomic percentages being 14.54%, 4.46%, 24.22%, 1.35%, 10.66%, and 44.77%, respectively. These results verify the successful synthesis of the CaBiO_2_Cl/g-C_3_N_4_ composite in this study.

#### 2.1.4. HR-TEM-EDS

**Figure 4:**
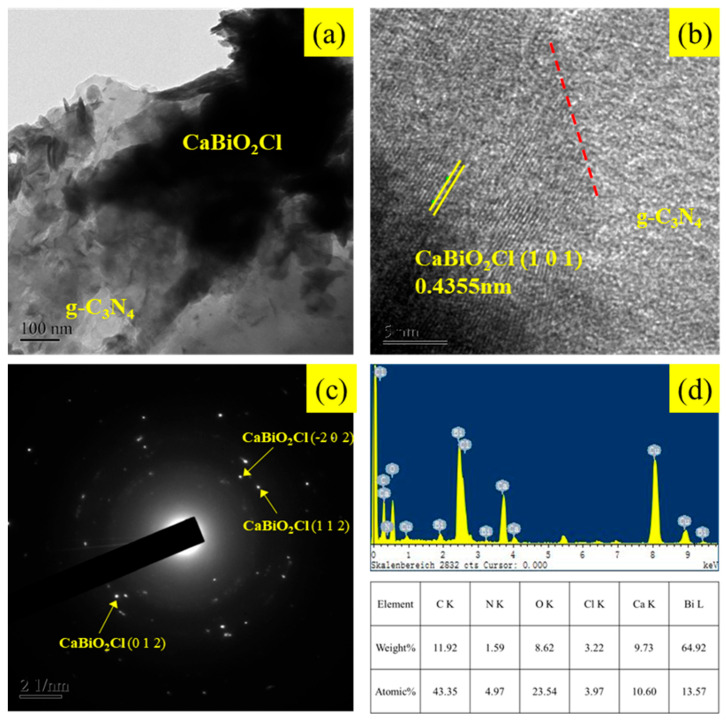
(**a**) FE−TEM images, (**b**) HR−TEM, (**c**) SAD, and (**d**) EDS of CaBiO_2_Cl/10 wt%g-C_3_N_4_.

The heterojunction interface of the composite sample was analyzed using high-resolution transmission electron microscopy (FE-TEM) equipped with an energy-dispersive X-ray spectrometer (EDS). Figure 4a presents a bright-field TEM image of the CaBiO_2_Cl/10 wt% g-C_3_N_4_ composite, where the light gray region in the lower-left corner corresponds to g-C_3_N_4_, while the dark black region represents CaBiO_2_Cl. Figure 4b shows a high-resolution TEM image of the composite, revealing lattice fringes with a *d*-spacing of 0.4355 nm, which corresponds to the (101) diffraction plane of CaBiO_2_Cl, confirmed through XRD analysis. Figure 4c displays the selected area electron diffraction (SAED) pattern of CaBiO_2_Cl/g-C_3_N_4_, revealing identifiable diffraction peaks corresponding to the (−202), (112), and (012) planes of CaBiO_2_Cl. The diffuse diffraction ring originates from g-C_3_N_4_, which exhibits lower crystallinity, consistent with the broad characteristic peaks observed in the XRD pattern. Figure 4d presents the EDS analysis results, confirming the presence of C, N, O, Cl, Ca, and Bi in an atomic ratio of 43.35:4.97:23.54:3.97:10.60:13.57, further supporting the successful formation of the CaBiO_2_Cl/g-C_3_N_4_ heterojunction. The Cu signal originates from the carbon-coated copper grid used in the TEM analysis. Figure S1 presents the elemental mapping images, illustrating the distribution of each element. The results show that C is uniformly distributed, while Bi, O, Ca, and Cl share the same distribution region, confirming the successful formation of the CaBiO_2_Cl/g-C_3_N_4_ composite.

#### 2.1.5. X-Ray Photoelectron Spectroscopy Spectra

**Figure 5:**
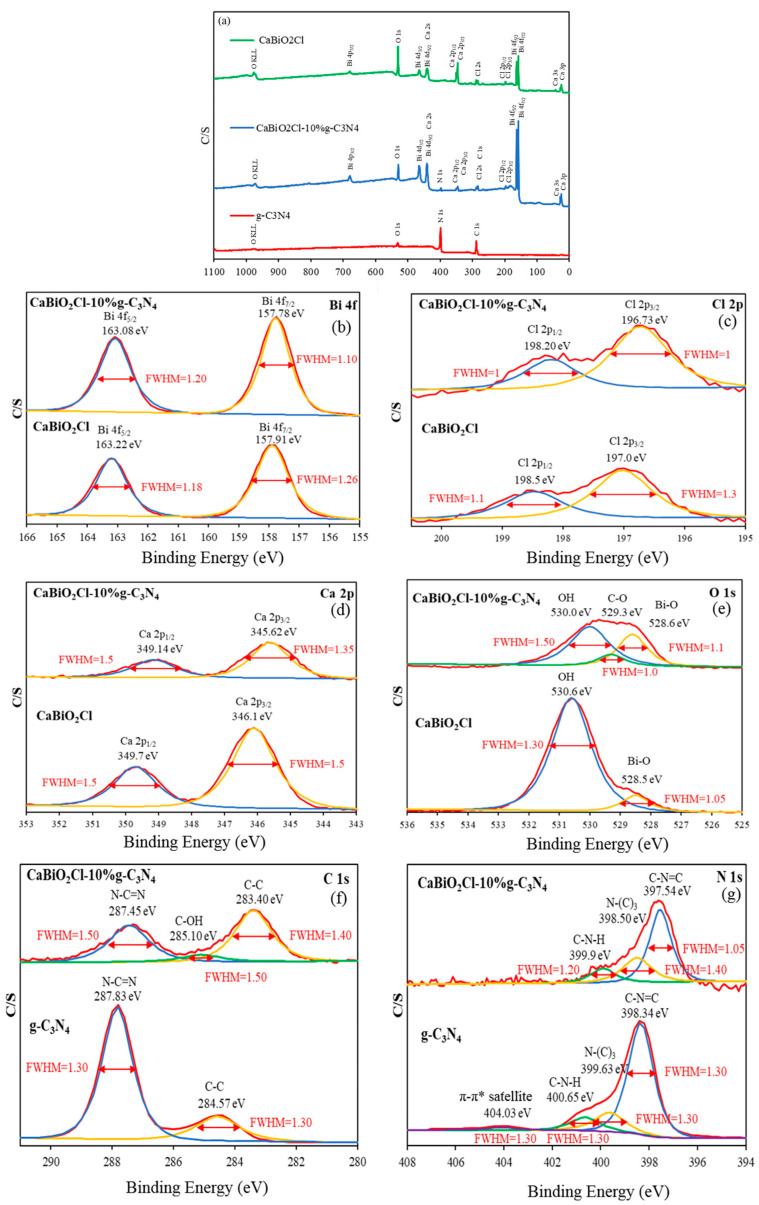
HR-XPS results for the as-prepared CaBiO_2_Cl, g-C_3_N_4_, and CaBiO_2_Cl/10 wt%-g-C_3_N_4_ samples, (**a**) survey, (**b**) Bi 4f, (**c**) Cl 2p, (**d**) Ca 2p, (**e**) O 1s, (**f**) C 1s, and (**g**) N 1s.

Using high-resolution X-ray photoelectron spectroscopy (HR-XPS), core electrons were excited through high-energy X-ray irradiation, allowing for the analysis of the elemental composition and valence states of CaBiO_2_Cl, CaBiO_2_Cl/10 wt% g-C_3_N_4_, and g-C_3_N_4_ (Figure 5a). The HR-XPS results confirm that CaBiO_2_Cl consists of four elements, Bi, Ca, O, and Cl, while g-C_3_N_4_ comprises C and N. In the case of CaBiO_2_Cl/10 wt%-g-C_3_N_4_, all six elements (Bi, Ca, O, Cl, C, and N) are present. The XPS spectrum of g-C_3_N_4_ clearly shows O 1s and O KLL Auger peaks. -OH groups are likely to replace amino groups (-NH_2_) on the surface of g-C_3_N_4_ nanoparticles [40]. Figure 5b shows the narrow spectra of Bi 4f for CaBiO_2_Cl and CaBiO_2_Cl/10 wt% g-C_3_N_4_, displaying two main characteristic peaks. The binding energies of pure CaBiO_2_Cl are 157.91 eV and 163.22 eV, while those for CaBiO_2_Cl/10 wt% g-C_3_N_4_ are 157.78 eV and 163.08 eV, corresponding to Bi 4f_7/2_ and Bi 4f_5/2_, respectively. The narrow spectrum of Cl 2p also exhibits two main characteristic peaks (Figure 5c). The binding energies of pure CaBiO_2_Cl are 197.00 eV and 198.50 eV, whereas those of CaBiO_2_Cl/10 wt% g-C_3_N_4_ are approximately 196.73 eV and 198.20 eV, corresponding to Cl 2p_3/2_ and Cl 2p_1/2_, respectively. Figure 5d shows the narrow spectrum of Ca 2p, presenting two main characteristic peaks. The binding energies of pure CaBiO_2_Cl are 346.10 eV and 349.70 eV, while those of CaBiO_2_Cl/10 wt%-g-C_3_N_4_ are 345.62 eV and 349.14 eV, corresponding to Ca 2p_3/2_ and Ca 2p_1/2_, respectively. Figure 5e presents the narrow spectrum of O 1s, which shows that the binding energy of the Bi-O bond contribution in pure CaBiO_2_Cl appears at 528.50 eV, while the O-H peak appears at 530.60 eV. In CaBiO_2_Cl/5 wt%-g-C_3_N_4_, the Bi-O bond contribution appears at 528.60 eV, the C-O bond contribution at 529.30 eV, and the O-H peak at 530.00 eV. Compared to other peaks, the peak corresponding to the O-H bond has a higher intensity, which may be attributed to the presence of surface moisture [41]. Figure 5f shows the narrow spectra of C 1s for g-C_3_N_4_ and CaBiO_2_Cl/10 wt%-g-C_3_N_4_; after peak fitting, it reveals three sub-peaks in CaBiO_2_Cl/10 wt%-g-C_3_N_4_, corresponding to N-C=N (287.45 eV), C-OH (285.10 eV), and C-C (283.40 eV) bonds [42]. In contrast, g-C_3_N_4_ exhibits two sub-peaks corresponding to N-C=N (287.83 eV) and C-C (284.57 eV) bonds [43]. Additionally, significant differences in the number and intensity of peaks between CaBiO_2_Cl/10 wt% g-C_3_N_4_ and g-C_3_N_4_ confirm that the changes in the chemical state of C 1s are closely related to the composite formation. Figure 5g presents the narrow spectra of N 1s. After peak deconvolution, CaBiO_2_Cl/10 wt%-g-C_3_N_4_ exhibits three characteristic peaks, all corresponding to g-C_3_N_4_, which are assigned to C–N–H, N–(C)_3_, and C–N=C bonds, with binding energies of 399.20 eV, 398.10 eV, and 397.20 eV, respectively. In contrast, g-C_3_N_4_ exhibits four characteristic peaks after deconvolution, attributed to π-π* satellite peaks, C–N–H, N–(C)_3_, and C–N=C bonds, with binding energies of 404.03 eV, 400.65 eV, 399.63 eV, and 398.34 eV, respectively [44].

#### 2.1.6. Ultraviolet–Visible Diffuse Reflectance Spectroscopy

The optical absorption properties of the photocatalyst samples were investigated using UV–Vis diffuse reflectance spectroscopy (UV–Vis DRS). The experimental data were analyzed with the Kubelka–Munk equation (Equation (1)).

αhν=A(hν − Eg)^n/2^ (1)

**Figure 6:**
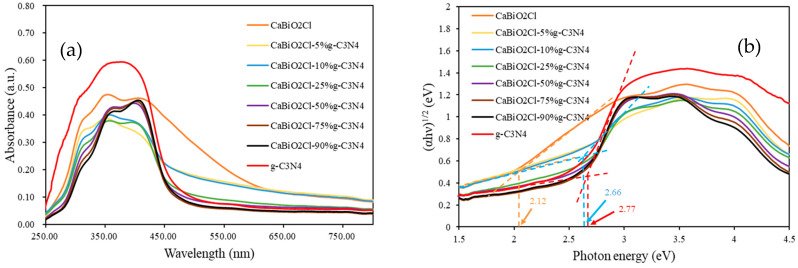
(**a**) Diffusion reflectance curves under different g-C_3_N_4_ contents and (**b**) Tauc plot of as-prepared CaBiO_2_Cl/g-C_3_N_4_ from diffuse reflectance spectra.

Equation (1) presents “α” as the absorption coefficient, “h” as Planck’s constant, “ν” as the photon frequency, “A” as a proportionality constant, and “Eg” as the bandgap energy. The Tauc method was further applied to convert the data into Tauc plots, from which the optical bandgaps were determined. The results revealed that the bandgap of CaBiO_2_Cl is approximately 2.12 eV, while that of g-C_3_N_4_ is about 2.77 eV. With the incorporation of g-C_3_N_4_, the bandgaps of the composites lie between those of the two pristine components. Figure 6 presents the DRS spectra of CaBiO_2_Cl photocatalysts with different g-C_3_N_4_ loadings. Notably, the bandgap of CaBiO_2_Cl/10 wt% g-C_3_N_4_ was estimated to be 2.66 eV, indicating that g-C_3_N_4_ incorporation effectively modulates the electronic structure, enabling visible-light excitation of valence band (VB) electrons into the conduction band (CB) and thereby facilitating the photocatalytic process.

#### 2.1.7. S_BET_

**Figure 7:**
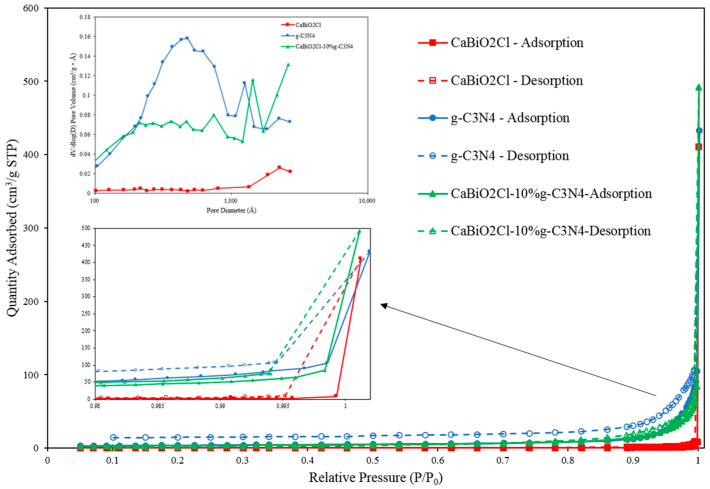
Nitrogen adsorption–desorption isotherms and the corresponding pore size distribution curve (inset) for CaBiO_2_Cl,g-C_3_N_4_ and CaBiO_2_Cl/g-C_3_N_4._

The specific surface area, pore size, and pore volume of CaBiO_2_Cl/10 wt%-g-C_3_N_4_ were measured using a surface area analyzer via the nitrogen adsorption–desorption isothermal method and compared with those of pristine CaBiO_2_Cl and g-C_3_N_4_. Figure 7 shows the nitrogen adsorption–desorption isotherms for CaBiO_2_Cl, CaBiO_2_Cl/10 wt%-g-C_3_N_4_, and g-C_3_N_4_, respectively. All samples exhibited type-III isotherms with H3 hysteresis loops according to the IUPAC classification [45], characterized by a convex shape typical of type-III adsorption. The specific surface areas of CaBiO_2_Cl, CaBiO_2_Cl/10 wt%-g-C_3_N_4_, and g-C_3_N_4_ were 0.6865, 11.9745, and 12.9017 m^2^/g, respectively, while their corresponding pore volumes were 0.0156, 0.0986, and 0.1344 cm^3^/g. Pore size distributions were determined using the Barrett–Joyner–Halenda (BJH) method. The pore characteristics indicate mesoporous structures (P/P_0_ > 0.8, pore size = 2–50 nm), consistent with the FE-SEM observations. As summarized in Table 1, the specific surface area and pore size of the composite fall between those of the individual CaBiO_2_Cl and g-C_3_N_4_, whereas its pore volume is relatively smaller.

### 2.2. Photocatalytic Reaction Activity

#### 2.2.1. Photodegradation of Rh6G

**Figure 8:**
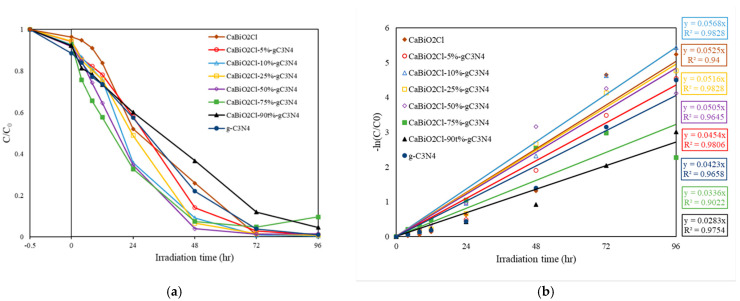
(**a**,**b**) Photocatalytic degradation of Rh6G as a function of irradiation time over CaBiO_2_Cl/g-C_3_N_4_.

The target pollutant degraded in this experiment is Rh6G dye. A visible-light photocatalytic experiment was conducted using 0.05 g of CaBiO_2_Cl, CaBiO_2_Cl/g-C_3_N_4_ composites (with 5%, 10%, 25%, 50%, 75%, and 90% g-C_3_N_4_), and g-C_3_N_4_ individually in 100 mL of 10 ppm Rh6G solution. Before the reaction, the system was kept in the dark for 30 min to ensure adsorption–desorption equilibrium of the catalyst. Concentration sampling was performed at fixed time intervals (4 h, 8 h, 12 h, 24 h, 48 h, 72 h, and 96 h), and the collected samples were analyzed using a UV-PDA to evaluate the photocatalytic performance of the synthesized catalysts and determine the optimal composite condition. Figure 8a presents the photocatalytic degradation efficiency of the catalysts. Additionally, the degradation rate was obtained using the first-order kinetics equation (Equation (2)), leading to Figure 8b, which shows the degradation rate curve and provides the *k* value and R^2^ value.

(2) $$\frac{dC_{t}}{dt}=-kC_{t}\to ln\frac{C_{t}}{C_{0}}=-kt$$

where *k* is the reaction rate constant (h^−1^), *C*_0_ is the initial concentration after the darkroom phase, *C_t_* is the concentration of the Rh6G solution varying with reaction time, and *t* is the reaction time (h).

From Figure 8a, it can be observed that the sample with 10 wt% g-C_3_N_4_ exhibits better photocatalytic performance compared to other composite ratios. As shown in Figure 8b, for composite ratios ranging from 0 to 100 wt%, the corresponding *k* values are 0.0525 h^−1^, 0.0454 h^−1^, 0.0568 h^−1^, 0.0516 h^−1^, 0.0505 h^−1^, 0.0336 h^−1^, 0.0283 h^−1^, and 0.0423 h^−1^, respectively, all with R^2^ values exceeding 0.90. Table 2 presents the overall Rh6G degradation efficiency for different CaBiO_2_Cl/g-C_3_N_4_ composite ratios. The *k* value of pure CaBiO_2_Cl is 0.0525 h^−1^ and that of pure g-C_3_N_4_ is 0.0423 h^−1^, while the *k* value of the CaBiO_2_Cl/10 wt% g-C_3_N_4_ composite increases to 0.0568 h^−1^, which is 1.1 and 1.3 times higher, respectively.

**Figure 9:**
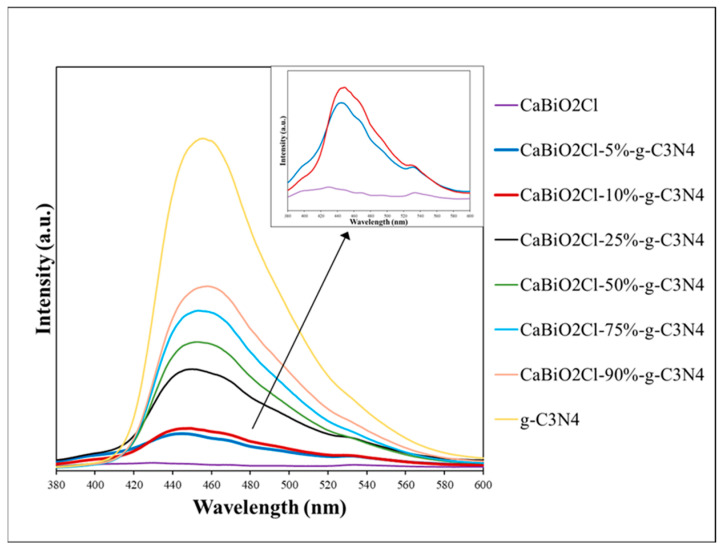
Photoluminescence spectra of as-prepared CaBiO_2_Cl/g-C_3_N_4_.

The differences in catalytic results may be related to factors like the specific surface area, pore size, or pore volume of the catalyst and the electron–hole recombination rate. In this study, fluorescence spectroscopy was employed to evaluate the electron–hole recombination behavior of the photocatalyst. Upon photon absorption, e^−^/h^+^ pairs are generated, initiating redox reactions that produce hydroxyl radicals and superoxide anion radicals essential for photocatalysis. However, if the electron–hole recombination rate is high, fewer charge carriers participate in redox reactions, reducing the generation of radicals and ultimately affecting photocatalytic efficiency. In the fluorescence spectra, higher intensity indicates a higher recombination rate of the catalyst. Figure 9 presents the fluorescence spectroscopy results for CaBiO_2_Cl, CaBiO_2_Cl/*x* wt% g-C_3_N_4_ (*x* = 5, 10, 25, 50, 75, 90), and g-C_3_N_4_. The results show that the composite samples significantly improved the high electron–hole recombination rate issue observed in pure g-C_3_N_4_. Furthermore, as the proportion of CaBiO_2_Cl increased, the effect became more pronounced, indicating that the composite photocatalyst effectively reduces the recombination rate and enhances photocatalytic performance, achieving the desired composite effect.

**Figure 10:**
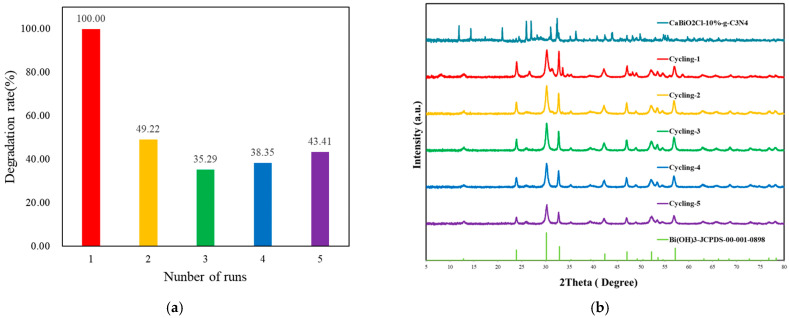
(**a**) Cycle runs and (**b**) XRD patterns acquired before and after the photocatalytic degradation of Rh6G in the presence of CaBiO_2_Cl/10 wt%g-C_3_N_4_.

Although the CaBiO_2_Cl/10 wt% g-C_3_N_4_ composite exhibits good initial photocatalytic activity, its recyclability remains unsatisfactory. A noticeable decline in catalytic performance was observed after several photocatalytic cycles (Figure 10a). This phenomenon may be attributed to several factors. First, reaction intermediates or byproducts may strongly adsorb on the catalyst surface, blocking the active sites and thus hindering subsequent reactions. Second, prolonged light irradiation may lead to partial photocorrosion or microstructural changes in the material, which can be detected through XRD analysis (Figure 10b). In addition, during the recovery process, fine catalyst particles may be lost due to poor sedimentation or low centrifugation efficiency, especially in suspension systems. Furthermore, repeated use may result in particle aggregation, leading to a decrease in specific surface area and reduced accessibility of active sites. These factors collectively contribute to the poor recyclability of the photocatalyst.

#### 2.2.2. CO_2_ Photoreduction Performance

**Figure 11:**
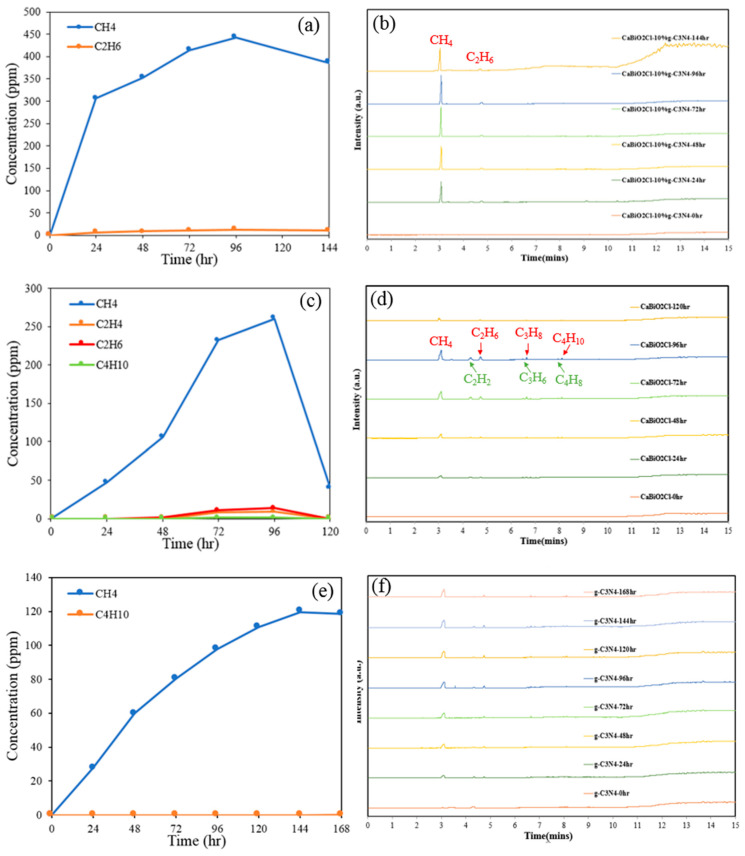
(**a**,**b**) Chromatograms of CaBiO_2_Cl/10 wt% g-C_3_N_4_, (**c**,**d**) CaBiO_2_Cl, and (**e**,**f**) g-C_3_N_4_ photocatalytic reduction of CO_2_ and as a function of irradiation time.

This study investigates the ability of the CaBiO_2_Cl/10 wt%g-C_3_N_4_ composite photocatalyst to reduce carbon dioxide and produce hydrocarbons, comparing its performance with the individual CaBiO_2_Cl and g-C_3_N_4_ photocatalysts to determine whether the composite exhibits enhanced reduction capability. CO_2_ is introduced into a sodium hydroxide solution, forming HCO_3_^−^, which adsorbs onto the catalyst surface through the photocatalytic reaction. Subsequent hydrogenation, electron transfer, or coupling processes lead to the formation of hydrocarbons, alcohols, acids, and other organic compounds. The products in this study were analyzed using gas chromatography (GC), and the retention time (Rt) of CH_4_ was recorded at 2.945 min. In the interval between 4 and 5 min, peaks corresponding to C_2_H_4_, C_2_H_2_, and C_2_H_6_ were observed. Additional signals appeared at 6.393, 6.488, 7.847, 7.951, 9.336, and 9.403 min, which were attributed to C_3_H_6_, C_3_H_8_, C_4_H_8_, C_4_H_10_, C_5_H_10_, and C_5_H_12_, respectively [46]. Figure 11a,c,e illustrates the yield of various organic compounds produced via photocatalytic CO_2_ reduction by CaBiO_2_Cl, g-C_3_N_4_, and CaBiO_2_Cl/10 wt%-g-C_3_N_4_. Figure 11b,d,f presents the chromatographic spectra of these photocatalysts over time. The results indicate that the primary organic products generated by all three photocatalysts are alkanes, with methane being the dominant product. Some organic products had concentrations below the detection limit. Table 3 presents the alkane concentrations obtained from CO_2_ photoreduction using CaBiO_2_Cl, g-C_3_N_4_, and CaBiO_2_Cl/10 wt%-g-C_3_N_4_. The composite CaBiO_2_Cl/10 wt%-g-C_3_N_4_ exhibited a significantly higher alkane concentration than the individual photocatalysts. The methane yields of CaBiO_2_Cl/10 wt% g-C_3_N_4_, CaBiO_2_Cl, and g-C_3_N_4_ were 0.5652, 0.3331, and 0.1020 μmol·g^−1^·h^−1^, respectively, with corresponding selectivities of 97.19%, 94.35%, and >99%, as shown in Table 3. These results confirm that the formation of the composite photocatalyst can effectively enhance photocatalytic performance and that g-C_3_N_4_ exhibits an improved CO_2_ adsorption capacity, enabling more CO_2_ molecules to accumulate on the catalyst surface, which in turn raises the local CO_2_ concentration and enhances the efficiency of photocatalytic reduction [23]. In comparison with other Bi-based bimetallic catalysts, such as Cd_0.46_Bi_1.36_O_2_Br, which mainly produces CO during CO_2_ reduction with a yield of 36.3 μmol g^−1^ [47], the performance appears superior to that of CaBiO_2_Cl/10 wt% g-C_3_N_4_. However, experimental studies often employ different types of reactors without providing detailed information regarding the light intensity entering the reactor or the amount of catalyst used. Moreover, many reports do not calculate the quantum yield, making it difficult to achieve reliable comparisons, even though the results are generally reported in units of mmol g^−1^ h^−1^ or mmol g^−1^ [23]. In addition, most CO_2_ reduction products reported in the literature are CO, HCOOH, and CH_3_OH, whereas in our study hydrocarbon products were obtained with high selectivity toward CH_4_.

### 2.3. Mechanisms of Rh6G Photodegradation and CO_2_ Photoreduction

**Figure 12:**
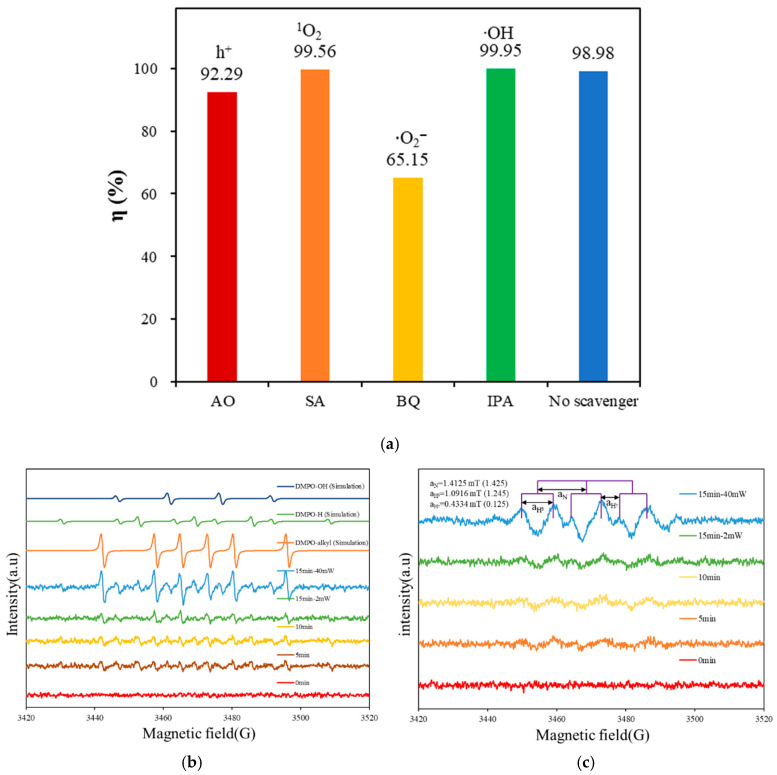
(**a**) The Rh6G concentration during photocatalytic degradation as a function of visible- light-driven irradiation time observed in CaBiO_2_Cl/10 wt% g-C_3_N_4_ under the addition of four scavengers: AO, SA, BQ, and IPA. (**b**,**c**) DMPO spin trapping EPR spectra for DMPO-•OH and DMPO-•O_2_^−^ under visible-light irradiation with CaBiO_2_Cl/10 wt% g-C_3_N_4_ photocatalyst.

To confirm the primary free radicals generated during the photocatalytic reaction, different radical scavengers were introduced during the degradation process. If the addition of a scavenger alters the photocatalytic performance, it can be inferred that the captured radical is the main active species produced by the synthesized catalyst. In this experiment, isopropanol (IPA), benzoquinone (BQ), ammonium oxalate (AO), and sodium azide (SA) were used as scavengers for hydroxyl radicals (∙OH), superoxide anion radicals (^•^O_2_^−^), holes (h^+^), and singlet oxygen (^1^O_2_), respectively. Figure 12a presents the concentration changes of the dye in the presence of different scavengers for the CaBiO_2_Cl/10 wt%-g-C_3_N_4_ system. The Y-axis (η) represents [(C_0_ − C)/C_0_] × 100%. It was observed that the removal rate of Rh6G decreased to 65.15% when benzoquinone (BQ) was added. Compared to other scavengers, BQ significantly inhibited the degradation process. This suggests that the primary reactive species involved in the photocatalytic degradation of Rh6G by CaBiO_2_Cl/10 wt%-g-C_3_N_4_ is the superoxide anion radical (^•^O_2_^−^).

During the photocatalytic process, photogenerated electrons and holes react with water and oxygen in the solution to produce ∙OH and ^•^O_2_^−^ radicals. To verify the presence of these radicals, 5,5-dimethyl-1-pyrroline *N*-oxide (DMPO) was used as a radical trapping agent. DMPO was mixed with different solutions and exposed to light to induce radical formation. The resulting adducts were detected using an EPR spectrometer, providing evidence of radical generation. A 150 W xenon lamp was used as the light source, and EPR measurements were conducted at 0 min, 5 min, 10 min, and 15 min of irradiation. Figure 12b,c display the EPR spectra of the CaBiO_2_Cl/10 wt%-g-C_3_N_4_ sample in a water and methanol solution containing DMPO. This analysis was used to determine whether ^•^O_2_^−^ radicals were produced during the photocatalytic reaction (Figure 12 c). No signal was detected in the dark. However, after 5 min of illumination, six characteristic peaks of ^•^O_2_^−^ appeared (a_N_ = 1.4125 mT, a_Hβ_ = 1.0916 mT, a_Hγ_ = 0.4334 mT). As the irradiation time increased, the intensity of these peaks became more pronounced. The topmost signal in the spectrum corresponds to an increased magnetic field (40 mW) at 15 min for enhanced signal interpretation. These findings confirm the presence of ^•^O_2_^−^ radicals during the photocatalytic reaction.

To discuss the photocatalytic reaction mechanism of the photocatalyst in this experiment, the bandgap energy of the photocatalyst was first determined using ultraviolet-visible diffuse reflectance spectroscopy (UV-Vis DRS). Additionally, ultraviolet photoelectron spectroscopy (UPS) was used to measure the *E*_VB_ values of the photocatalyst samples. The valence band energy of CaBiO_2_Cl was found to be 1.48 eV, while that of g-C_3_N_4_ was 1.62 eV. By substituting these values into Equation (3),

*E*_CB_ = *E*_VB_ − *E*g (3)

The *E*_CB_ values of the catalyst samples were calculated. Finally, these values were combined with the results of reactive species detection.

**Figure 13:**
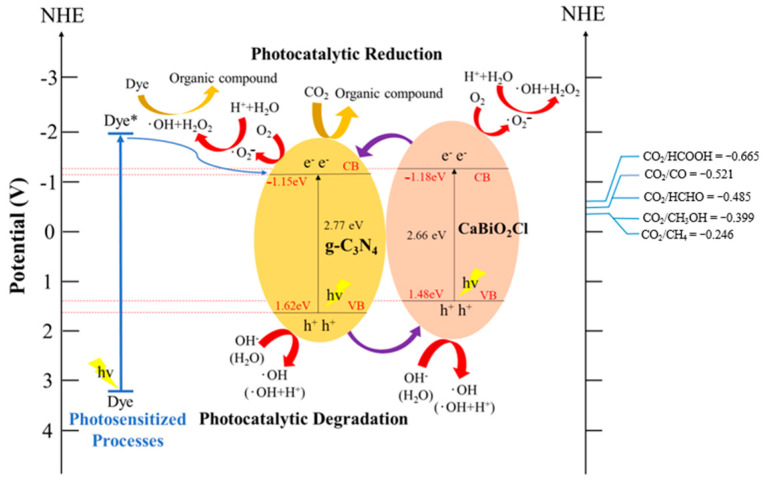
Schematic of the bandgap structures of CaBiO_2_Cl/10 wt% g-C_3_N_4_.

Figure 13 illustrates the electron–hole transfer mechanism of the CaBiO_2_Cl/10 wt%-g-C_3_N_4_ photocatalyst in the degradation of Rh6G dye. Upon visible-light irradiation, electrons are excited from the valence band to the conduction band, where they react with O_2_ to generate superoxide anion radicals (^•^O_2_^−^). Meanwhile, the holes (h^+^) remaining in the valence band oxidize OH^−^ and H_2_O to form hydroxyl radicals (∙OH), which subsequently degrade the target pollutant, Rhodamine 6G dye.

In the photocatalytic CO_2_ reduction experiments, CO_2_ dissolves in the NaOH solution to form multiple carbonate species (HCO_3_^−^, CO_3_^2−^, and H_2_CO_3_, depending on the pH). These species adsorb onto the catalyst surface through physical or chemical interactions prior to the photocatalytic reaction. Upon the introduction of the photocatalyst, these carbon species underwent a sequence of proton-coupled electron transfer (PCET) and dehydration processes, resulting in the formation of reactive surface-bound intermediates [48]. We previously employed a series of PbBiO_2_X photocatalysts and, by integrating photochemical performance data with in situ Raman spectroscopy, were able to clearly observe the surface of the photocatalysts and the distinct spectral features corresponding to key intermediates under different applied potentials. Distinct spectral features corresponding to key intermediates, such as *COO^−^, *CO, HCO/HCOH, *OCCO, *OCCHO, and C–H-containing species, were predominantly observed on the photocatalyst surface [49,50,51]. During the catalytic process, the competitive adsorption of *CO plays a pivotal role in determining the efficiency of C–C bond formation. The surface coverage of *CO is considered a critical descriptor for predicting the generation of C_2+_ and higher-order carbon products. Compared to the formation of C_1_ products, the production of C_2+_ species involves more complex electron transfer pathways and multi-step reaction mechanisms, with the C–C coupling step generally identified as the rate-determining step. This inherently introduces greater kinetic barriers to the process. Enhancing the chemical interaction between CO_2_ molecules and the photocatalyst surface is regarded as a key strategy for improving photocatalytic activity. In terms of the reaction mechanism, most carbon-based products are generated through sequential hydrogenation and dehydration steps, ultimately leading to the formation of terminal –CH_3_ groups. When adjacent –CH_2_ moieties are present on the surface, their coupling and subsequent desorption may give rise to alkene-type hydrocarbons. Alternatively, the coupling and elimination of –CH groups may result in the formation of alkyne-type hydrocarbons. In cases where no coupling or desorption of intermediates occurs, the predominant final product is expected to be methane, which serves as the primary target product in this study (see Figure S2) [46,52].

## 3. Materials and Methods Section

### 3.1. Chemicals

The following analytical-grade reagents were employed without additional purification: Bi(NO_3_)_3_·5H_2_O (Acros, 98%), CaCO_3_ (Shimakyu, 98%), KCl (Shimakyu, 99.8%), melamine (Alfa Aesar, 99.0%), Rh6G (TOKYO Kasei Kogyo Co., Ltd., 99%), ammonium oxalate (AO; Shimakyu, 99.0%), *p*-benzoquinone (BQ; Alfa Aesar, 98.0%), sodium azide (SA; Sigma-Aldrich, 99.5%), isopropanol (IPA; Merck, 99.9%), and NaOH (Shimakyu, 95%).

### 3.2. Analytical Instruments and Methods

A variety of instruments were employed for spectroscopy and microscopy analyses. X-ray diffraction (XRD) patterns were collected using a Rigaku SmartLab diffractometer (Tokyo, Japan) with Cu-Kα radiation at 40 kV and 80 mA. High-resolution transmission electron microscopy (HR-TEM), selected area electron diffraction (SAED), and electron-dispersive spectroscopy (EDS) measurements were performed on a JEOL-2010 microscope (Tokyo, Japan) at 200 kV. Field-emission scanning electron microscopy (FE-SEM) combined with EDS was conducted using a JEOL JSM-7401F instrument (Tokyo, Japan) at an accelerating voltage of 15 kV. Ultraviolet–visible diffuse reflectance spectra (UV–Vis DRS, 300–800 nm) were recorded at room temperature with an SA-13.1 spectrophotometer (Scinco, Seoul, Republic of Korea). High-resolution X-ray photoelectron spectroscopy (HR-XPS) analyses were carried out using a Micromeritics Gemini system from ULVAC-PHI (Kanagawa, Japan). For CO_2_ photoreduction experiments, a Thermo Trace 1300 gas chromatograph (Thermo Fisher, Waltham, MA, USA) equipped with both flame ionization and thermal conductivity detectors was employed.

### 3.3. Synthesis of Photocatalysts

#### 3.3.1. Synthesis of CaBiO_2_Cl

CaBiO_2_Cl photocatalyst was synthesized using high-temperature solid-state calcination. First, 5 mmol of Bi(NO_3_)_3_·5H_2_O was dissolved in 25 mL of ethanol to prepare solution A. Separately, 5 mmol of KCl was dissolved in 10 mL of distilled water to form solution B. Solutions A and B were then mixed and stirred at room temperature with a magnetic stirrer for 4 h. The solid precipitate was separated through filtration and thoroughly rinsed with deionized water and ethanol to eliminate residual molecular or ionic species, and we dried BiOCl under vacuum at 60 °C for 12 h.

The CaBiO_2_Cl sample was synthesized through a solid-state reaction. Analytical-grade CaCO_3_ and BiOCl (1:1 molar ratio) were thoroughly ground in an agate mortar, placed in alumina crucibles, and calcined at 800 °C for 12 h in a muffle furnace. The product was then allowed to cool naturally to room temperature.

#### 3.3.2. Synthesis of g-C_3_N_4_

g-C_3_N_4_ powder was synthesized by calcining 5 g of melamine in a semi-closed alumina crucible with a lid at 540 °C for 4 h in a muffle furnace (heating rate: 10 °C/min) under ambient atmosphere. The resulting product was cooled to room temperature and collected as a yellow powder.

#### 3.3.3. Synthesis of CaBiO_2_Cl/g-C_3_N_4_

The preparation method of CaBiO_2_Cl/g-C_3_N_4_ involves taking *x* wt% of g-C_3_N_4_ (*x* = 5 wt%, 10 wt%, 25 wt%, 50 wt%, 75 wt%, 90 wt%) and y wt% of CaBiO_2_X, where x + y = 100%. Using 10 mL of ethylene glycol as the solvent, the mixture is placed in an autoclave and heated in an oven at 100 °C for 4 h. After cooling to room temperature, the product is filtered with 2 L of deionized water and then dried in an oven at 60 °C for 24 h. Finally, the dried material is ground using an agate mortar to obtain the CaBiO_2_Cl/g-C_3_N_4_ composite.

### 3.4. Photocatalysis Experiments

#### 3.4.1. Photocatalytic Degradation of Rh6G

To understand the photocatalytic degradation rate of the synthesized catalyst, 0.05 g of photocatalyst sample was weighed and added to 100 mL of 10 ppm Rh6G dye solution. The mixture was first stirred in the dark for 30 min to reach adsorption equilibrium. Then, a light irradiation experiment was conducted using an illumination chamber equipped with an 18 W visible-light lamp. Samples were taken at specific time intervals (4 h, 8 h, 12 h, 24 h, 48 h, 72 h, and 96 h) during the reaction process. For each sampling, 5 mL of the solution was withdrawn and centrifuged at 4000 rpm for 15 min. After the first centrifugation, 4 mL of the supernatant was taken for a second centrifugation, and, finally, 3 mL of the upper clear solution was collected as the test sample. The solution was analyzed using UV-PDA spectroscopy, with the absorbance of the dye measured at 530 nm to determine its concentration. When the absorbance of the standard solutions is plotted against their concentrations, a direct linear relationship should be observed. The linearity of this plot arises from the Beer–Lambert law (Equation (4)), which states that the absorption of light by a substance is proportional to its concentration in solution.

A = (εl)c (4)

Here, “A” represents the absorbance, “ε” denotes the molar extinction coefficient (M^−1^·cm^−1^), “l” is the length of the light path through the cuvette (cm), and “c” indicates the solution concentration (M).

The equation of the Beer–Lambert law represents a straight line, which generally takes the form of Equation (5).

*y* = m*x* + b (5)

where the slope “m” is equal to εl and the concentration “c” of the unknown solution can be determined by using its measured absorbance along with the slope of the best-fit line.

#### 3.4.2. Photocatalytic Reduction of CO_2_

First, 300 mL of a 1 M NaOH aqueous solution was prepared in a 500 mL quartz glass reactor. CO_2_ gas was then introduced at a flow rate of 500 L/min for 1 h to ensure full saturation. After ceasing the gas flow, a 1 mL gas sample was taken from the reactor and analyzed through gas chromatography (GC) to establish the background level. Next, 0.1 g of the catalyst was added, followed by stirring. The reactor was subsequently placed in a photoreactor chamber, and the light source was activated to begin the photocatalytic reaction. Gas sampling (1 mL) was carried out every 24 h, and the product yield was measured using GC. The illumination system consisted of sixteen ultraviolet lamps, each rated at 8 W, serving as the experimental light source.

## 4. Conclusions

In this study, a CaBiO_2_Cl/g-C_3_N_4_ heterojunction photocatalysts was successfully synthesized, and its photocatalytic performance under visible-light irradiation was systematically investigated for Rh6G degradation and CO_2_ reduction. The results showed that the composite containing 10 wt% g-C_3_N_4_ exhibited the highest activity in both reactions, significantly outperforming the individual components. This enhanced performance can be attributed to its superior light absorption, efficient charge carrier separation, and increased specific surface area and mesoporous structure, which provide more active sites and facilitate the generation of reactive species. Radical scavenging experiments confirmed that ^•^O_2_^−^ is the primary active species in Rh6G photodegradation. Under ambient conditions (1 atm, 25 °C), the CO_2_-to-CH_4_ photocatalytic conversion rate of CaBiO_2_Cl/g-C_3_N_4_ reached 0.5652 μmol g^−1^ h^−1^. These results demonstrate that the composite exhibits excellent photocatalytic activity and holds significant potential for the visible-light-driven degradation of organic pollutants and environmental remediation.

## Figures and Tables

**Table 1:** Surface area, pore volume, and pore size of the photocatalysts.

Sample	Surface Area (m^2^/g)	Pore Volume (cm^3^/g)	Pore Size (nm)
CaBiO_2_Cl	0.6865	0.0156	32.9464
CaBiO_2_Cl/10 wt% g-C_3_N_4_	11.9745	0.0986	26.8036
g-C_3_N_4_	12.9017	0.1344	34.1077

**Table 2:** Pseudo-first-order rate constants for the degradation of Rh6G with photocatalysts under visible-light irradiation.

	CaBiO_2_Cl							
g-C_3_N_4_ (wt%)	0	5	10	25	50	75	90	100
*k* (h^−1^)	0.0525	0.0454	0.0568	0.0516	0.0505	0.0336	0.0283	0.0423
R^2^	0.9400	0.9806	0.9828	0.9828	0.9645	0.9022	0.9754	0.9658

**Table 3:** CH_4_ yield and selectivity of CO_2_ reduction by different photocatalysts.

Photocatalyst	Concentration (ppm)					CH_4_ Yield	CH_4_ Selectivity
	CH_4_	C_2_H_6_	C_3_H_8_	C_4_H_10_	C_5_H_12_	(μmol·g^−1^·h^−1^)	(%)
CaBiO_2_Cl/10 wt% g-C_3_N_4_	441.99	12.81	---	---	---	0.5652	97.19
CaBiO_2_Cl	260.49	13.92	---	1.69	---	0.3331	94.35
g-C_3_N_4_	119.65	---	---	---	---	0.1020	>99%

## Data Availability

The original contributions presented in the study are included in the article; further inquiries can be directed to the corresponding author.

## Supplementary Materials

Supplementary data associated with this article can be found in the online version at: https://www.mdpi.com/article/10.3390/molecules30183760/s1. Figure S1. Mapping of the of CaBiO_2_Cl/10wt% g-C_3_N_4_ sample. Figure S2. Schematic illustration of CO_2_ photocatalytic conversion by catalysts.
