# Supplementary material for: Visible-Light-Driven Photocatalysis of Carbon Dioxide and Organic Pollutants by CaBiO2Cl/g-C3N4
Source: Molecules. 2025 Sep 16;30(18):3760. doi: 10.3390/molecules30183760 (PMC12472916; doi:10.3390/molecules30183760)
Supplement: Supplementary file 1 [file molecules-30-03760-s001.zip › molecules-3856903-supplementary.pdf]

Visible-Light-Driven Photocatalysis of Carbon Dioxide and Organic Pollutants by $CaBiO_2Cl/g-C_3N_4$

Yu-Yun Lin, Bo-Heng, Huang, Wen-Yu You, Fu-Yu Liu, Jia-Hao Lin, Chiing-Chang Chen*

Department of Science Education and Application, National Taichung University of Education, Taichung 403514, Taiwan

* Author to whom correspondence should be addressed

E-mail: ccchen@mail.ntcu.edu.tw

Fax: +886-4-2218-3560

Tel: +886-4-2218-3406

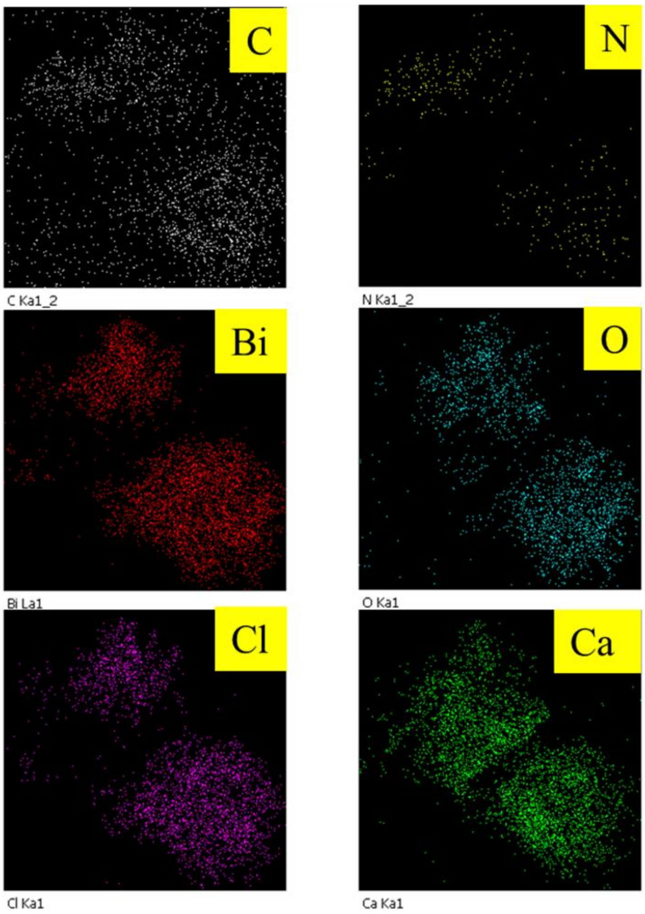

Figure S1. Mapping of the of $CaBiO_2Cl$/10wt% g-C_3N_4 sample.

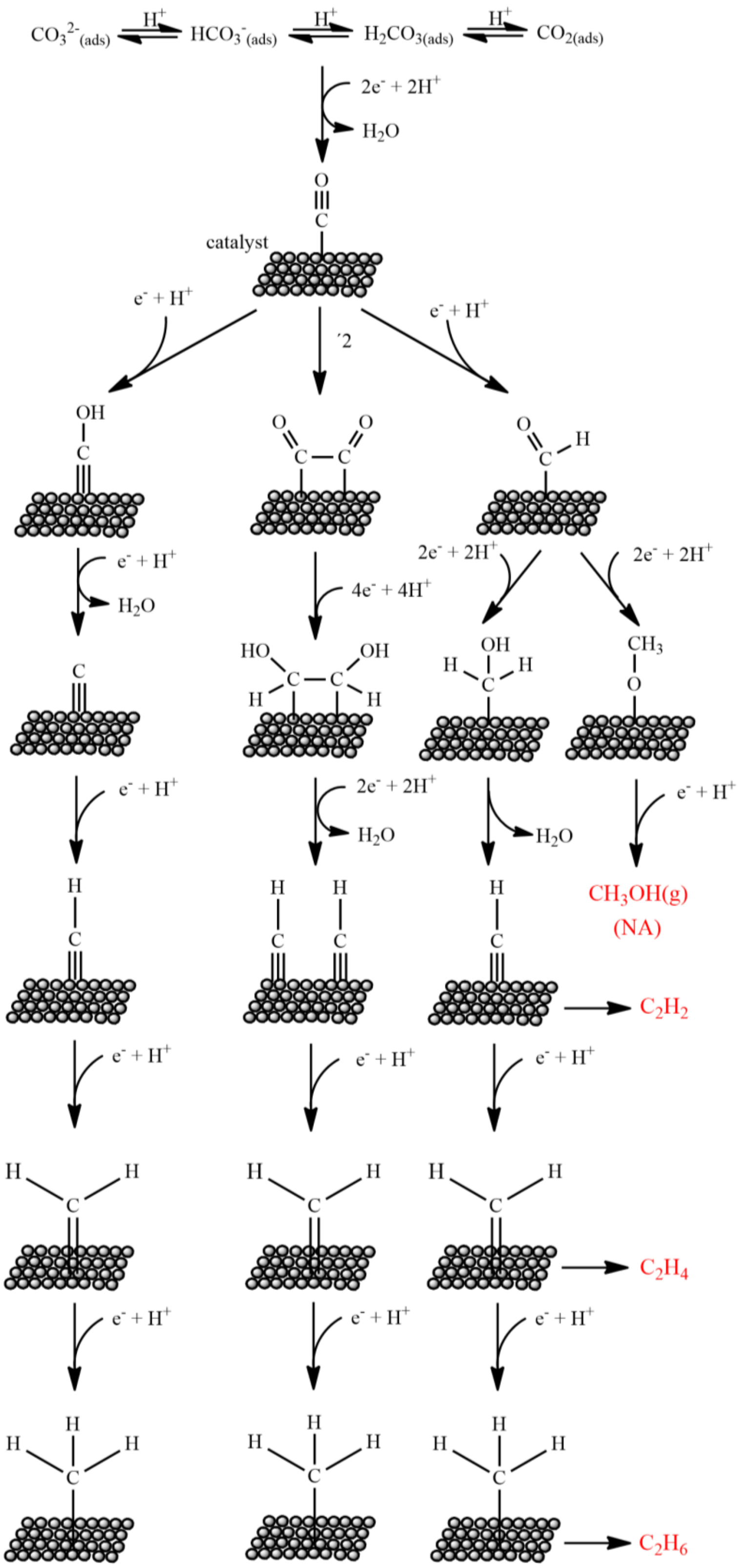

Figure S2. Schematic illustration of CO_2 photocatalytic conversion by catalysts.
